# Cerebral Angiography under Artificial Intelligence Algorithm in the Design of Nursing Cooperation Plan for Intracranial Aneurysm Patients in Craniotomy Clipping

**DOI:** 10.1155/2022/2182931

**Published:** 2022-07-11

**Authors:** Wenhui Xu, Yanan Xie, Xu Zhang, Wei Li

**Affiliations:** ^1^Operation Room, The First Affiliated Hospital of Zhejiang Chinese Medical University (Zhejiang Provincial Hospital of Traditional Chinese Medicine), Hangzhou, 310000 Zhejiang, China; ^2^Anesthesia Operation Center, Hainan Hospital of PLA General Hospital, Sanya, 572013 Hainan, China; ^3^Operation Room, Zhejiang Provincial People's Hospital, Hangzhou, 310000 Zhejiang, China

## Abstract

This research was to investigate the value of indocyanine green angiography (ICGA) based on maximum interclass variance (Otsu) method in the nursing plan of intracranial aneurysm clipping (ICAC) for intracranial aneurysm patients. An Otsu algorithm was selected to optimize the original images with the optimal threshold. In addition, the algorithm was applied to ICGA images of 86 patients with intracranial aneurysms, who were randomly divided into an experimental group (using ICGA + ICAC+ perioperative nursing) and a control group (ICAC + conventional nursing), to observe the clinical indicators, treatment, complications, nursing satisfaction, and quality of life of patients in two groups. The results showed that the mean square error (MSE), structural similarity (SSIM), and shape error (SE) were 3.71, 0.84, and 0.47, respectively. The length of hospital stay in the experimental group (19.9 ± 3.5 days) was significantly shorter than that in the control group (23.2 ± 3.0 days), the rate of excellent treatment was significantly higher than that in the control group, and the incidence of complications was lower. WHOQOL-BREF scores of the two groups after nursing intervention were higher than before, and the score in the experimental group was higher than the control group. In addition, the nursing satisfaction was also significantly higher in the experimental group, and the difference was statistically significant (*P* < 0.05). In conclusion, ICGA based on the Otsu method could effectively evaluate the cerebrovascular morphology during craniotomy and ICAP and improve the surgical efficacy. Combined with perioperative nursing intervention, it could greatly reduce the incidence of postoperative complications, improve the treatment effect and quality of life, and enhance the long-term prognosis.

## 1. Introduction

Intracranial aneurysm refers to the pathological bulge of the vascular wall caused by a series of pathophysiological changes in cerebral arteries. The rupture of aneurysm is the most common cause of subarachnoid hemorrhage, with a mortality of 25%-30% for the first ruptured hemorrhage, and the mortality of rebleeding is as high as 60%. The current epidemiological statistics show that the incidence of aneurysm in adults is about 1% [1, 2]. It is often discovered only because of clinical symptoms after rupture, so it is called an untimed bomb deep in the brain. The extremely high mortality and disability have undoubtedly increased people's attention to this disease [3].

At present, the surgical intervention methods for the treatment of intracranial aneurysms mainly consist of two surgical methods, which are microscopic intracranial aneurysm clipping (ICAC) and endovascular interventional embolization [4]. There are many important perforating vessels near the site of aneurysm, with large individual differences. Studies have found that endovascular interventional treatment of aneurysms brings a high recurrence and high cost. Therefore, ICAC under the microscope is still the major surgical intervention means [5]. Complete disappearance of the aneurysm, while maintaining normal blood flow to the parent and perforating vessels, are regarded as the sign of success of the surgery. However, related reports in recent years have shown that the residual rate of the tumor neck is as high as 10% after ICAC. Moreover, the incidence of vascular stenosis or occlusion caused by clipping is more than 15%. It has become a difficulty for many surgeons to improve this situation so as to promote the success rate of surgery [6, 7].

Indocyanine green angiography (ICGA) is an emerging intraoperative auxiliary operation in recent years. Indocyanine green (ICG) reagent is injected intravenously, and a high-power microscope with integrated near-infrared fluorescence imaging function is used to irradiate the surgical field of view. The direction of the main blood vessels can be observed in real time, the morphological characteristics of the aneurysm and its anatomical relationship with the parent artery can be distinguished, and the course of the nearby perforating arteries can be known. For the improvement of the surgical effect of ICAC, the residual of tumor neck is reduced, and the perforating vessels in the functional area are protected [8, 9]. Medical image segmentation is of great significance in medical image processing and analysis. It is the basis for a series of follow-up operations such as structure and motion analysis, three-dimensional visualization, disease diagnosis, treatment, evaluation, and virtual surgery as well as surgical guidance. It is also still the bottleneck in the development of medical image processing [10, 11].

The maximum between-class variance method (Otsu method) is an adaptive threshold determination method. Since it was proposed by the Japanese scholar Nobuyuki Otsu, it has been also called the Otsu method or the Otsu algorithm. It divides each pixel into two parts, background and target, according to the grayscale characteristics of the image. If the between-class variance between the background and the target is larger, it means that the grayscale difference between the two parts in the image is larger. When some parts of the target and the background are misclassified mutually, the difference between the two parts will be reduced. Thus, the maximum between-class variance means the smallest probability of being misclassified [12–14]. ICAC is one of the common methods for the treatment of intracranial aneurysms, but the surgery is traumatic to a certain extent. The recovery may be affected due to both physical and psychological trauma in patients. Therefore, it is extremely important to increase the efforts of nursing intervention for patients. In recent years, the use of perioperative nursing intervention has been proposed in clinical practice, which has achieved ideal results and improved the prognosis of patients effectively [15, 16].

Some neurosurgeons in the world have applied ICGA and perioperative nursing method to ICAC. However, there are few reports on whether the intraoperative application can reduce related postoperative complications and improve the prognosis and its shortcomings around the world. Meanwhile, there are also few scholars combine artificial intelligence algorithms in cerebral angiography [17]. Therefore, a cerebral angiography under the Otsu algorithm was adopted here to explore its therapeutic advantages and prognostic effects in the design of nursing cooperation plan for ICAC. It was expected to provide some theoretical guidance for the treatment and prognosis of ICAC in patients with tumors.

## 2. Materials and Methods

### 2.1. General Information

The clinical data of 86 patients with intracranial aneurysm who were treated in the hospital from April 2019 to August 2021 were taken. There were 39 males and 47 females, aged 49–61 years old with an average age of 53 years old. The patients were randomly divided into experimental group and control group (with 43 patients in each). In experimental group, ICAC with Otsu method-based ICGA under the microscope was performed together with perioperative nursing intervention. In control group, ICAC under the microscope and routine nursing intervention were given. All patients and their families included have signed the informed consents, and this study had been approved by ethics committee of hospital.

The inclusion criteria included the following. The patients were 18–75 years old, with no gender requirement. Through the imaging data, aortic arch computed tomography angiography (CTA) of the head and neck, or digital subtraction angiography (DSA), the aneurysm was confirmed in patients. All the included patients and their families took the craniotomy as the primary treatment method. The patients met the clinical guidelines that craniotomy to clip aneurysm was allowed.

The exclusion criteria were as follows. The patients had coagulation dysfunction or anaphylaxis to the contrast agent ICG. Patients were assessed with grade 5 in the Hunt-Hess grading. The patients suffered from dysfunction of important organs such as the heart, liver, lung, and kidney, or the patients were complicated with neurological diseases. Female patients were pregnant or breastfeeding.

### 2.2. Surgeries and Postoperative Treatments

The surgeries were all performed by senior and experienced chief physicians. The surgical method for patients in the control group was as follows. Intravenous-inhalation general anesthesia was given; after the patients were successfully anesthetized, the head frame was fixed and strictly sterilized. As the optimal surgical approach was taken, the proximal and distal ends of the aneurysm were shown. The arachnoid near the neck of the aneurysm was sharply cut, and the perforating vessels were protected. After clear separation, temporary blocking clips were used to clip the parent artery temporarily (<9 min). The most matching titanium clips were selected to clip the tumor neck according to the parent vessel, the shape of the aneurysm, and the width of the aneurysm neck. Papaverine solution was taken to soak the parent artery. It was observed whether the pulse of parent artery was abnormal, and the anesthesiologist was instructed to control the arterial systolic pressure in a range of 130-140 mmHg. After strict hemostasis, the dura mater was sutured, and 3-4 fixation clamps were used to fix bone flaps. The drainage tube was indwelled under the scalp, and the scalp was sutured layer by layer.

The surgical method for patients in the experimental group was basically the same as that in the control group. After careful separation and exposure of the aneurysm, the nurse was instructed to rapidly inject ICG solution at a dose of 0.5 mg/kg. The light was turned off, and the near-infrared angiography mode of the microscope was on. The Otsu method was adopted to process the images, and the direction of the main vessel as well as the position and width of the neck of the aneurysm was observed. The perforating vessels and parent artery were protected as possible, and the aneurysm was then clipped. For the blood supply of the parent artery and perforating artery, such as residual aneurysm neck, the angle or number of aneurysm clips should be changed. The surgical field was kept to be clear, and the bleeding was stopped in time. The angiography was repeated after 15 minutes, and the cranium was closed after the effect was satisfied, the same as the control group.

Patients in both groups were given neurointensive care after surgery. Head computerized tomography (CT) was reviewed within 1 hour after surgery to check for rebleeding or not. 24 hours after surgery, head CT was performed again to observe brain tissue edema. Comprehensive treatment was given, including the monitoring of intracranial pressure as well as water and electrolyte balance, infection prevention, fluid infusion, prevention of vasospasm, and drug treatment to prevent epilepsy. Cranial CTA/DSA was reexamined after surgery, to determine whether the neck of intracranial aneurysm was residual and whether perforating vessels were clipped. Examination of head magnetic resonance diffusion weighted imaging (DWI) was also performed to get to know whether there was a new cerebral infarction area.

### 2.3. Nursing Cooperation for Patients

In control group, routine nursing intervention was given, patients' health knowledge education was given, and various examinations during the perioperative period were improved. In addition, the development of the patient's condition was observed, and targeted treatment was given if complications occurred.

In experimental group, before the surgery, the relevant diagnosis and treatment knowledge of the disease and the hospital environment was introduced to patients and their families, in order to comfort and encourage the patients to relieve their fears and nervousness. In addition, strictly monitor the general vital signs of patients, and disinfect all instruments and equipment in advance. During the surgery, it should disinfect the towel, master the operation technique, and assist the doctor to complete the operation smoothly. After the surgery, it should give the patient correct dietary guidance, instruct the patient to keep the pipeline unobstructed, assist the patient to turn over, arrange the sheets regularly, and keep them dry and clean. When various nursing was implemented, it was necessary to strictly follow the aseptic principle to prevent infection.

### 2.4. Otsu Method

It was assumed that the pixel values of a given image were divided into *K* gray levels, which were represented as *1*, *2*, *3*, *…*, *K*. The number of pixels with pixel gray level *i* was represented by *a*_*i*_, and the total number of pixels in the entire image was *A* = *a*_1_ + *a*_2_ + ⋯+*a*_*K*_. To simplify, the grayscale histogram normalization was expressed in the form of a probability distribution, which was shown as

(1) $$\begin{array}{c} q_{i}=\frac{a_{i}}{A},\\ q_{i}\geq 0,\\ \overset{K}{\underset{i=1}{\sum }}q_{i}=1. \end{array}$$

It was also assumed that a threshold *t* was used to divide the pixels of the image into two categories: target *B*_0_ and background *B*_1_. *B*_0_ represented the pixels containing the gray levels [1, ⋯, *t*], while *B*_1_ represented the pixels containing the gray levels [*t* + 1, ⋯, *t*]. Therefore, the probability and mean value of each type of events could be expressed as

(2) $$\begin{array}{c} \omega_{0}=Qr\left(B_{0}\right)=\overset{t}{\underset{i=1}{\sum }}q_{i}=\omega \left(t\right), \end{array}$$

(3) $$\begin{array}{c} \omega_{0}=Qr\left(B_{1}\right)=\overset{K}{\underset{i=t+1}{\sum }}q_{i}=1-\omega \left(\text{t}\right), \end{array}$$

(4) $$\begin{array}{c} \mu_{0}=\overset{t}{\underset{i=1}{\sum }}iQr\left(i\left|B_{0}\right.\right)=\overset{t}{\underset{i=1}{\sum }}\frac{iq_{i}}{\omega_{0}}=\frac{\mu \left(t\right)}{\omega \left(t\right)}, \end{array}$$

(5) $$\begin{array}{c} \mu_{1}=\overset{K}{\underset{i=t+1}{\sum }}iQr\left(i\left|B_{1}\right.\right)=\overset{K}{\underset{i=t+1}{\sum }}\frac{iq_{i}}{\omega_{1}}=\frac{\mu_{E-}\mu \left(t\right)}{1-\omega \left(t\right)}. \end{array}$$

In the above equations, the following equations (6) and (7) were workable.

(6) $$\begin{array}{c} \omega \left(t\right)=\overset{t}{\underset{i=1}{\sum }}q_{i}, \end{array}$$

(7) $$\begin{array}{c} \mu \left(t\right)=\overset{t}{\underset{i=1}{\sum }}{iq}_{i}. \end{array}$$

*ω*(*t*) and *μ*(*t*) are the zero-order and first-order cumulative statistical distances of the image histogram when the gray levels were *1* to *t*, respectively; and equation (8) was workable.

(8) $$\begin{array}{c} \mu_{E}=\mu \left(K\right)=\overset{K}{\underset{i=1}{\sum }}{iq}_{i}. \end{array}$$

This was the overall mean value of the entire image. Thus, when the threshold *t* was any value, the equation (9) was obtained.

(9) $$\begin{array}{c} \omega_{0}\mu_{0}+\omega_{1}\mu_{1}=\mu_{E},\\ \omega_{0}+\omega_{1}=1. \end{array}$$

The variance of the two classes of target and background were expressed as equations (10) and (11), respectively.

(10) $$\begin{array}{c} \sigma_{0}^{2}=\overset{t}{\underset{i=1}{\sum }}{\left(i-\mu_{0}\right)}^{2}Qr\left(i\left|B_{0}\right.\right)=\overset{t}{\underset{i=1}{\sum }}\frac{{\left(i-\mu_{0}\right)}^{2}q_{i}}{\omega_{0}}, \end{array}$$

(11) $$\begin{array}{c} \sigma_{1}^{2}=\overset{K}{\underset{i=t+1}{\sum }}{\left(i-\mu_{1}\right)}^{2}Qr\left(i\left|B_{1}\right.\right)=\overset{K}{\underset{i=t+1}{\sum }}\frac{{\left(i-\mu_{1}\right)}^{2}q_{i}}{\omega_{1}}. \end{array}$$

In the equation (10), *σ*_0_^2^ represented the target variance, *ω*_0_ was the zero order when it was 0, and *μ*_0_ was the cumulative statistical distance when it was 0. In equation (11), *σ*_1_^2^, *ω*_1_, and *μ*_1_ stood for the background variance, the zero order when it was 1, and the cumulative statistical distance when it was 1.

To determine whether a threshold segmentation was suitable or not, a series of evaluation criteria are needed to be formulated for discriminant analysis. The following standard of equation (12) was used in the Otsu threshold selection algorithm.

(12) $$\begin{array}{c} \lambda =\frac{\sigma_{C}^{2}}{\sigma_{V}^{2}},\\ \kappa =\frac{\sigma_{E}^{2}}{\sigma_{V}^{2}},\\ \eta =\frac{\sigma_{C}^{2}}{\sigma_{E}^{2}}. \end{array}$$

In the equation above, equations (13) and (14) were workable.

(13) $$\begin{array}{c} \sigma_{V}^{2}=\omega_{0}\sigma_{0}^{2}+\omega_{1}\sigma_{1}^{2}, \end{array}$$

(14) $$\begin{array}{c} \sigma_{C}^{2}=\omega_{0}{\left(\mu_{0}-\mu_{E}\right)}^{2}+\omega_{1}{\left(\mu_{1}-\mu_{E}\right)}^{2}=\omega_{0}\omega_{1}{\left(\mu_{1}-\mu_{2}\right)}^{2}. \end{array}$$

The equation (15) was then worked out according to equation (9).

(15) $$\begin{array}{c} \sigma_{E}^{2}=\overset{K}{\underset{i=1}{\sum }}{\left(i-\mu_{E}\right)}^{2}q_{i}. \end{array}$$

The intraclass variance, the between-class variance, and the total variance of the overall image when the gray level was *t* were represented, respectively. In such a situation, the optimal threshold selection could be simplified to finding the value of threshold *t*. Thus, it satisfied any one of the criteria to obtain the maximum value in equation (12), and these three criteria were equivalent to each other. As the evaluation standards were set, the threshold could be satisfied that it maximized the degree of separation between the target and the background. This threshold was just the optimal threshold to be selected.

The basic relationship was maintained by the above three variances, and the relationship was expressed as

(16) $$\begin{array}{c} \sigma_{V}^{2}+\sigma_{C}^{2}=\sigma_{E}^{2}. \end{array}$$

*σ*_*V*_^2^ and *σ*_*C*_^2^ were the functions of the threshold *t*, while *σ*_*E*_^2^ was independent of the threshold *t*. Also, *σ*_*V*_^2^ was based on second-order statistics, while *σ*_*C*_^2^ was on the basis of first-order statistics. Therefore, *η* became the simplest one out of the three above-mentioned threshold selection criteria. The optimal threshold *t*^∗^ was the *t* value that made *η* had the maximum value in the gray levels, which was equivalent to the *t* value satisfied that *σ*_*C*_^2^. took the maximum value. According to equations (6) and (7), equations (17) and (18) were obtained.

(17) $$\begin{array}{c} \eta \left(t\right)=\frac{\sigma_{C}^{2}\left(t\right)\;}{\sigma_{E}^{2}}, \end{array}$$

(18) $$\begin{array}{c} \sigma_{C}^{2}\left(t\right)=\frac{{\left[\mu_{E}\omega \left(t\right)-\mu \left(t\right)\right]}^{2}}{\omega \left(t\right)\left[1-\omega \left(t\right)\right]}. \end{array}$$

The optimal threshold *t*^∗^ was the *t* value that satisfied equation (19). If the maximum value of *σ*_*C*_^2^(*t*) at this time corresponded to multiple *t* values, the average of these *t* values was taken as *t*^∗^.

(19) σC2t∗=σC2t1≤t≤Kmax.

The Otsu algorithm flow could be described as the following steps.

- The histogram of the input image was computed and normalized. *Q*_*i*_ and *i* = 1, 2, ⋯, *K* were taken to represent each component of each gray level of the histogram
- Equation (2) was computed with *i* = 1, 2, ⋯, *K*, and then the cumulative sum *ω*_0_ was calculated
- Equation (4) was computed with *i* = 1, 2, ⋯, *K*, and the cumulative mean *μ*_0_ was calculated
- The global grayscale mean value *μ*_*E*_ of the image was calculated with equation (8).
- Equation (18) was computed with *i* = 1, 2, ⋯, *K*, and the between-class variance *σ*_*C*_^2^(*t*) was calculated
- The optimal threshold *t*^∗^ was selected, that was, the *t* value that satisfied the maximum value of *σ*_*C*_^2^(*t*). If *t* was not the only in this case, the average value was taken

### 2.5. Observation Indicators

The operation time, intraoperative blood loss, and the length of hospital stay of patients in the experimental group and the control group were counted. The recurrence and prognosis of the two groups were observed after 6 months of follow-ups. According to the Glasgow outcome scale (GOS), the prognosis was evaluated. GOS had a total score of 15, in which 4 points for eye opening response, 5 points for verbal response, and 6 points for motor response. In eye opening response, 4 points were scored for automatic eye opening, 3 points for eye opening as being called, 2 points for pain-induced eye opening, and 1 point for noneye opening. In verbal response, 5, 4, 3, 2, and 1 point were for normal orientation, wrong responses, speech errors, indiscernible speech, and no speech, respectively. In motor response, 6, 5, 4, 3, 2, and 1 point indicated being able to act according to commands, localizing pain, avoiding stinging pain, flexing the limb with stinging pain, hyperextending the limb with stinging pain, and no motion, respectively. The highest GOS score was 15 points, and the lowest was 3. The lower the score, the more severe the disease. Usually, the disease was more likely to recover with a GOS score of 8 or more. The prognosis was generally poorer if the GOS score was less than 7. Patients are potentially at risk of death with the GOS score of 3-5 and accompanied with a loss of brainstem reflexes [18]. The patients could take care of themselves, the symptoms disappeared, or there were mild neurotoxicity symptoms, and the prognosis was assessed as good. The patients needed be cared for in their life, or they were in a vegetative state, and the symptoms of severe neurotoxicity still existed, and the prognosis was poor.

The *World Health Organization Quality of Life* (WHOQOL-BREF) was adopted to evaluate the quality of life of patients in the two groups before and after nursing intervention. This scale was composed of 4 dimensions, including physical health, mental health, social ability, and environmental adaptability. Each dimension had a score ranging from 0 to 100 points [19]. The higher the score, the higher the quality of life of the patient. The types and incidence of complications in both groups were observed and compared, and the complications included rerupture of aneurysm, cerebral vasospasm, hydrocephalus, and cerebral infarction. Nursing satisfaction of patients in the two groups was counted. 90-100 points, 80-90 points, 70-80 points, and less than 70 points indicated very satisfied, satisfied, basically satisfied, and dissatisfied, respectively.

### 2.6. Statistical Analysis

SPSS 22.0 software was used for statistical analysis of all data. The rate (%) was used to express enumeration data, and *χ*^2^ was used for the test of enumeration data. The measurement data were represented by mean ± standard deviation ($\bar{\text{x}}\pm s$), and the independent samples *t*-test was used for comparison of measurement data between the two groups. When *P* < 0.05, it meant that the difference of data between the two groups was statistically significant.

## 3. Results

### 3.1. General Information

For patients in the experimental group, there were 24 males and 19 females. They aged 52-65 years old, with an average age of 53.0 years old. In term of Hunt-Hess grading, 9 cases were of grade I, 13 cases of grade II, 15 cases of grade III, and 6 cases of grade IV. In the aneurysm location, 11 cases had anterior communicating aneurysm, 7 cases had posterior communicating aneurysm, 5 cases had internal carotid aneurysm, 13 cases had middle cerebral aneurysm, and 7 cases had basilar aneurysm. In the morphology of aneurysm, small aneurysm (<5 mm) was observed in 8 cases, medium-sized aneurysm (5-10 mm) was in 17 cases, large aneurysm (10-25 mm) was in 12 cases, and giant aneurysm (>25 mm) was in 6 cases.

**Figure 1:**
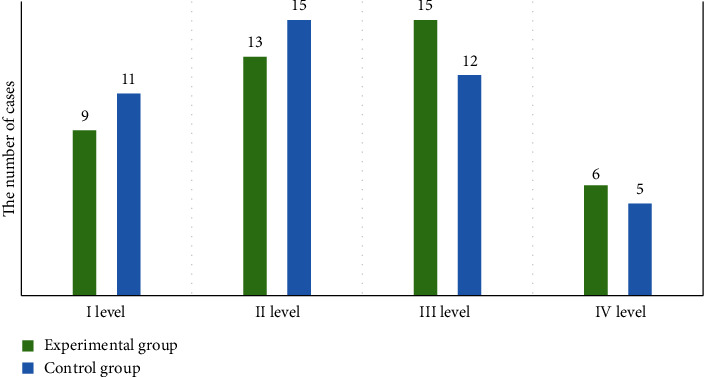
Hunt-Hess grading of patients in two groups.

**Figure 2:**
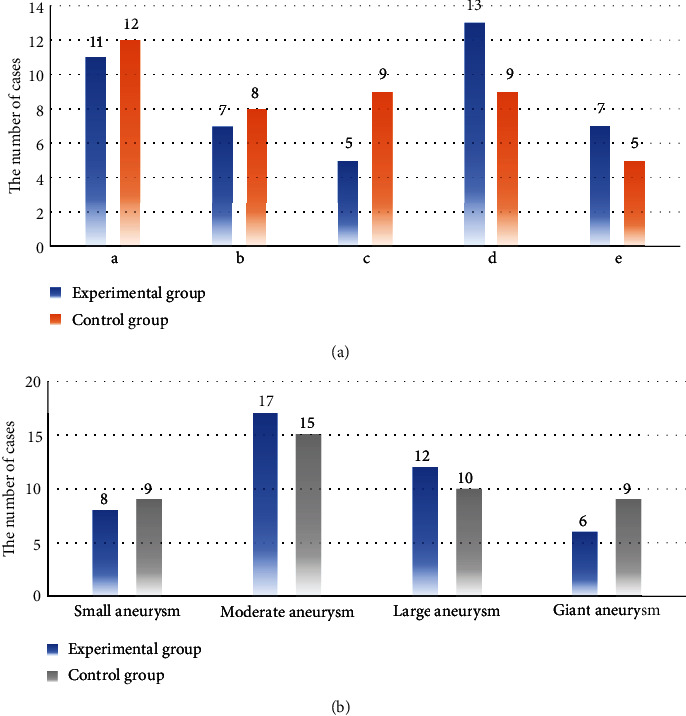
The location and morphology of the aneurysm in patients in the two groups. (a) represented the location of aneurysms in patients in the two groups (A, B, C, D, and E stood for anterior communicating aneurysm, posterior communicating aneurysm, internal carotid aneurysm, middle cerebral aneurysm, and basilar aneurysm, respectively); (b) represented the aneurysm morphology in patients.

In the control group, there were 20 male patients and 23 female patients, with the age of 53-64 years old and the average age of 53.0 years old. With Hunt-Hess grading, 11, 15, 12, and 5 cases were of grade I, grade II, grade III, and grade IV, respectively. For the aneurysm location, 12, 8, 9, 9, and 5 cases got anterior communicating aneurysm, posterior communicating aneurysm, internal carotid aneurysm, middle cerebral aneurysm, and basilar aneurysm. For aneurysm morphology, 9, 15, 10, and 9 cases had the small aneurysm (<5 mm), medium-sized aneurysm (5-10 mm), large aneurysm (10-25 mm), and giant aneurysm (>25 mm). More details are shown in Figures 1 and 2.

### 3.2. Segmentation and Reconstruction Results of Cerebral Angiography

**Figure 3:**
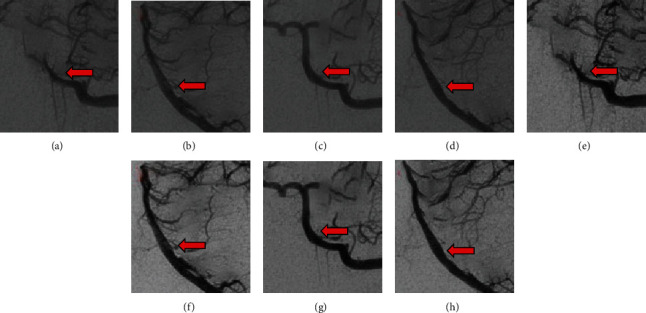
Cerebral angiography images of the patient with aneurysm. (a–d) were the images before processing by Otsu method, while (e–h) were those images processed by Otsu method. The arrows indicated the location of the aneurysm in each mage.

**Figure 4:**
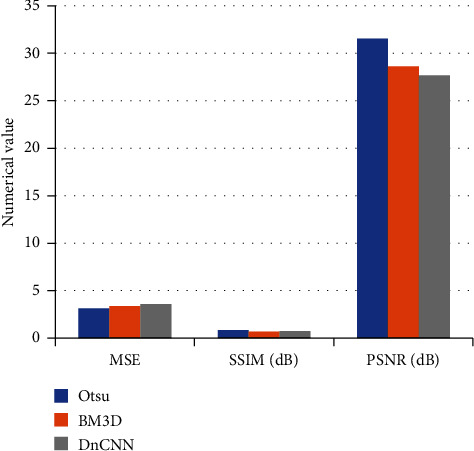
Comparison of the segmentation and reconstruction results of cerebral angiography.

Aneurysm cerebral angiography images of patients were shown before and after processing by the Otsu method. The mean square error (MSE), structural similarity (SSIM), and shape error (SE) were 3.71, 0.84, and 0.47, respectively, after processing, which are represented in Figures 3 and 4 for details.

### 3.3. Perioperative Clinical Indicators

**Figure 5:**
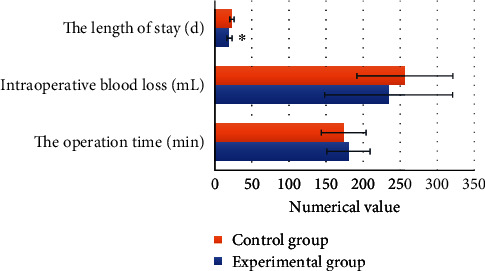
Perioperative clinical indicators of patients in two groups. ∗Compared with control group, *P* < 0.05.

The operation time of the experimental group and the control group was 180.3 ± 29.2 minutes and 173.9 ± 30.3 minutes, respectively. The intraoperative blood loss was 234.4 ± 86.4 mL and 256.4 ± 64.7 mL, respectively; the length of hospital stay was 19.9 ± 3.5 days and 23.2 ± 3.0 days, respectively. Compared with that of the control group, the length of hospital stay of patients in the experimental group was significantly shorter as *P* < 0.05, indicating the difference was statistically significant, while there was no significant difference in the operation time and intraoperative blood loss between the two groups for *P* > 0.05 (Figure 5).

### 3.4. Clinical Treatment Effect

**Figure 6:**
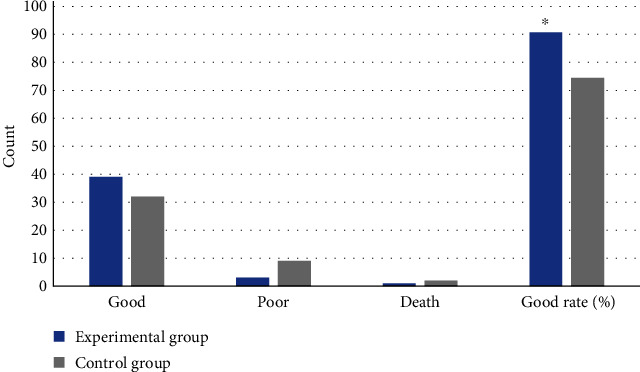
Clinical treatment effect of two groups. ∗Compared with that of control group, *P* < 0.05.

In the treatment effect of the experimental group and the control group, 39 cases and 32 cases were assessed to be good, respectively; and 3 cases and 9 cases got poor effect, respectively. 1 case and 2 cases died in the experimental group and the control group, respectively; the good rates were 90.70% and 74.42%, respectively. The good rate of the experimental group was significantly higher than that of the control group as *P* < 0.05, so the difference was statistically significant.  Figure 6 gives more details.

### 3.5. Incidence of Complications

**Figure 7:**
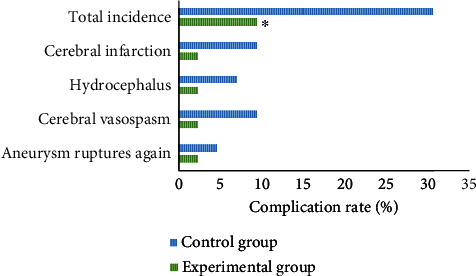
The incidence of complications of the two groups. ∗Compared with control group, *P* < 0.05.

There were 4 cases and 13 cases that got complications in the experimental group and the control group, respectively; the total incidence was 9.30% and 30.23%, respectively. The total incidence of complications in the experimental group was significantly lower than that in the control group (*P* < 0.05), with the statistically significant difference. Details could be found in Figure 7.

### 3.6. Comparison of the Scores of Each Dimension of WHOQOL-BREF

**Figure 8:**
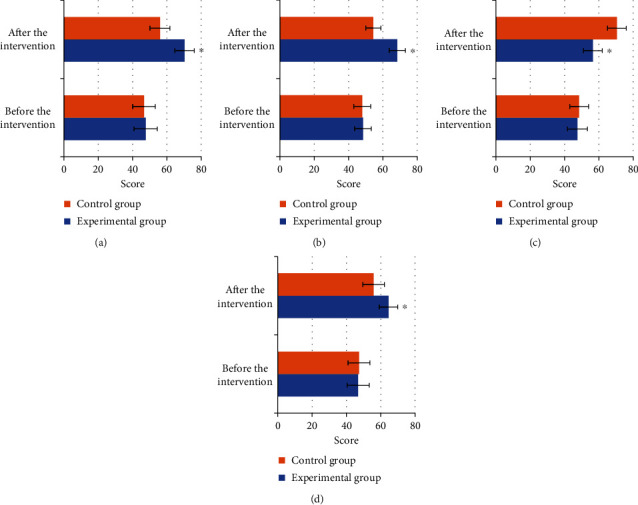
Comparison of WHOQOL-BREF scores before and after nursing intervention in the two groups. (a–d) showed the physical health score, mental health score, social ability score, and environmental adaptability score, respectively. ∗Compared with those of control group, *P* < 0.05.

The quality of life of patients in the two groups was evaluated before and after nursing intervention, using WHOQOL-BREF. After the nursing intervention, the physical health, mental health, social ability, and environmental adaptability of patients in both groups were significantly improved compared with those before nursing. The improvement in the experimental group was significantly higher than that in the control group; *P* < 0.05, so the differences were statistically significant. It is shown in Figure 8 for details.

### 3.7. Nursing Satisfaction

**Figure 9:**
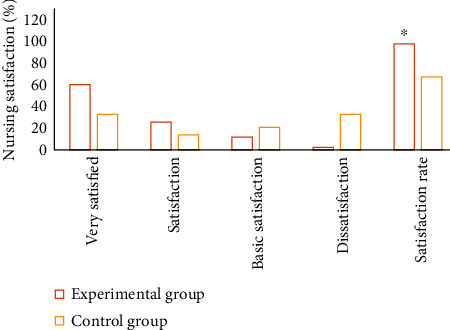
Nursing satisfaction of two groups. ∗ Compared with that of control group, *P* < 0.05.

With statistical analysis of the nursing satisfaction of patients in the two groups, it was found that the nursing satisfaction rate of the experimental group was significantly higher than that of the control group as *P* < 0.05, which suggested that the difference was statistically significant.  Figure 9 displays the nursing satisfaction for details.

## 4. Discussion

Subarachnoid hemorrhage caused by rupture of intracranial aneurysm has a sudden onset and rapid progression, often with a sudden increase in intracranial hematoma in a short period of time. It leads to irreversible damage to the nervous system, resulting in serious sequelae and even threat to life [20, 21]. Intracranial aneurysms are often located at the bifurcations of blood vessels, and the blood has unequal shear stress on the vessel wall, which makes the aneurysms easy to form [22].

In the 1960s, microscopic craniotomy to clip aneurysm was first performed in foreign countries. Since then, the sign of a successful surgery has been recognized as complete disappearance of the aneurysm, as well as the maintaining of normal blood flow to both parent and perforating vessels. But up to now, human beings have invested a lot of material and financial resources to improve the safety and effectiveness of aneurysm treatment [23]. After ICAC, the residual tumor neck, the collateral damage to parent blood vessels, and the incidence of serious complications remain high. It has been pointed out through some studies that intraoperative application of ICGA is an ideal choice for craniotomy, because the application of microscopic infrared fluorescence imaging technology during the surgery allows the relatively complete morphological structure of the exposed blood vessels on the surface of the brain to be observed through a microscope [24, 25]. Therefore, it is believed that the application of ICGA in neurosurgery may be helpful for evaluating intraoperative cerebral vascular morphology, choosing to clip aneurysm, and reducing residual neck of the aneurysm.

The clinical data of 86 patients who underwent ICAC were statistically analyzed, and there was no statistical significance in the general data as *P* > 0.05. ICGA + ICAC can be performed for any intracranial aneurysm, but the imaging of basilar aneurysms is not as clear as that of anterior circulation aneurysms, which may be related to the complex and diverse anatomical structure as well as the deep location. No significant difference was found in the operation time and intraoperative blood loss between the experimental group and the control group (*P* > 0.05). This was consistent with the findings of Park et al. [26]. The reason was that although ICGA was performed during ICAC, it took some time to wait; so there were some differences in the surgical procedure. However, the surgeons had rich experience and accurate acquirement of the location and orientation of the aneurysm with complete preoperative examinations. The intraoperative angiographic field was clear, and most of the parent artery and perforating blood vessels were completely exposed. During the intraoperative angiography, the bleeding was fully stopped, so there was no difference in the operation time between the two groups.

It was discovered that there was a statistically significant difference in the length of hospital stay between the experimental group and the control group, which was similar to the findings of Hao and Wei [27]. The reasons might be related to the occlusion or stenosis of the perforating vessels and parent vessels during the surgery, which resulted in functional ischemia and secondary cerebral infarction. It was also found that the intracranial aneurysm patients who underwent ICAC under cerebral angiography had the significantly higher good rates of treatment than that in the control group, and the incidence of complications was significantly lower than that of the control group. These suggested that ICAC under cerebral angiography had a significant effect in the treatment of intracranial aneurysm for patients. This was conducive to reducing the economic burden of patients, reducing the risk of complications, and promoting the treatment of patients. Besides, the independent survival rate scored under the modified Rankin scale in the experimental group was significantly higher than that in the control group after 6 months of follow-ups, similar to the results of Matas et al. [28].

According to relevant literature, nursing intervention refers to the use of certain nursing procedures by nursing staff to conduct a comprehensive judgment and analysis of the patient's condition and take timely and effective measures in advance in the process of nursing to avoid complications [29]. The principle of nursing intervention is to prevent possible problems first and treat them once the problems occur. It ensures the life safety of patients and reduces the incidence of medical disputes and medical malpractices [30]. The results showed that the quality of life and nursing satisfaction of patients in the experimental group were significantly higher than those in the control group after treatment with nursing intervention in the perioperative period.

## 5. Conclusion

To sum up, the traditional microscopic ICAC of intracranial aneurysms and the routine nursing were compared with the ICGA images processed by Otsu method combined with perioperative nursing intervention. The latter could reduce the incidence of postoperative complications significantly, enhance the treatment effect effectively, promote the quality of life, and improve the long-term prognosis. Furthermore, ICGA was cheap, took less time to operate, and could be operated for multiple times with a high accuracy. Therefore, intraoperative ICGA, with the broad application prospects, was suitable for popularization and application in most hospitals. However, for the small sample size and the short postoperative follow-up time, some complications of ICAC usually would occur 3-5 years after surgery. These deficiencies would motivate further researches.

## Data Availability

The data used to support the findings of this study are available from the corresponding author upon request.
